# Social representations and interface layout: A new way of enhancing persuasive technology applied to organ donation

**DOI:** 10.1371/journal.pone.0244538

**Published:** 2020-12-31

**Authors:** Mathilde Barbier, Ladislav Moták, Camille De Gasquet, Fabien Girandola, Nathalie Bonnardel, Grégory Lo Monaco

**Affiliations:** 1 Social Psychology Laboratory, Aix-Marseille University, Aix-en-Provence, France; 2 Center for Research on the Psychology of Knowledge, Language and Emotion (PsyCLÉ), Aix-Marseille University, Aix-en-Provence, France; 3 ADEF, Aix-Marseille University, Marseille, France; Wright State University, UNITED STATES

## Abstract

Although campaigns promoting organ donation have proved their effectiveness, increasing the number of people who explicitly agree to become donors is still difficult. Based on the social marketing notion of persuasive technology, we reasoned that it was timely to focus on the design of this persuasive technology and to analyze its contribution in particularly challenging contexts such as organ donation. More specifically, the originality of the present study lay in the way we linked the field of persuasive technology to the theory of social representations, and combined them with an analysis of the ergonomic aspects of interface layout. This study had two complementary goals. The first was to determine whether the sociocognitive salience of the central elements of social representations (i.e., the most frequent and important themes related to the subject—here, organ donation—for individuals), can be used to achieve persuasive outcomes. The second was to determine whether interface layout, in terms of information location and background characteristics (color and contrast), can strengthen the persuasive impact. University students (*N* > 200) were exposed to a computer screen displaying a message involving either central or peripheral elements of the social representations of organ donation (status), placed either in the middle or on one side of the screen (location), and shown against either a white or a blue background (background). Eye-tracking data were recorded, in addition to self-reported data. In line with the elaboration likelihood model, results showed that participants who were exposed to central (vs. peripheral) elements of the social representations of organ donation followed the central route in processing information. Moreover, they had stronger attitudes, and more of them stated that they were *actual* organ donors. Importantly, however, at least for some variables, these status-related effects were not independent of the interface layout. More specifically, the persuasive impact of the central elements was enhanced when the information was displayed in the middle (vs. the side) of the screen and when it was displayed on a white (vs. blue) background. We discuss the theoretical and practical issues raised by these results.

## Introduction

Since the advent of the Internet in the 1970s and the subsequent expansion of websites in the 1990s and 2000s, digital media have gradually invaded our daily lives [1, 2]. In the present study, we explored whether digital interfaces can be designed as tools, based on the assumption that screens are not viewed passively, but are the locus of a genuine cognitive and ultimately human experience. We asked whether we can build on the success of digital development to design messages that individuals view as relevant to the topic being addressed.

We explored this issue in the challenging context of organ donation. Although transplantation is now commonplace, improving practices [3] is not enough to reduce the shortage of donor organs [4, 5]. Efforts must therefore be made to identify the barriers and levers in the decision to donate. Thus far, a common goal of the many published studies (for reviews, see [6–9]) has been to provide information and recommendations on organ donation. Reducing contradictions and ambiguities about organ donation is indeed important both for the families of deceased persons and for the general public. Moreover, knowing how to deliver the most appropriate information can help decision-makers and authorities build more effective campaigns to promote organ donation. This is precisely what we sought to do in the present study.

More specifically, we reasoned that persuasive technology can make a valuable contribution in our domain of interest (i.e., organ donation). First coined by Fogg [10], the term *persuasive technology* refers to a computer-enhanced opportunity to shape humans' attitudes and behaviors to achieve beneficial ends. This is possible because systems featuring human-machine interactions are able to combine the positive attributes of both interpersonal communication and mass communication to disseminate information to a wide audience [11, 12]. Importantly, research has shown that technology is able to serve many social issues, especially in the field of health (e.g., [13–16]), where technology can be used to motivate people to adopt healthy behaviors [17]. Here, we therefore focused on improving the design of persuasive technology and, more specifically, on its use in promoting organ donation.

Our research problem was therefore to persuade individuals to change their attitude toward organ donation and enable them to explicitly become *organ donors*. Persuasive processes are the result not of computers and technologies, but of human intention, and we therefore referred to the elaboration likelihood model (ELM) [18, 19]. The ELM helps to predict whether, in a given context, message content will be cognitively deeply processed or only superficially read. In the present study, we specifically assumed that (1) attitude change is guided by personal involvement [20], and that (2) this involvement in turn depends mainly on how the main argument of the message is framed. We further reasoned that this framing interacts with certain features and properties of the human-machine interaction. Two well-established and influential avenues of research are relevant here. First, the theoretical field of social representations can be used to guide the elaboration of the message’s content. Second, the development of persuasive technology requires work on the medium (i.e., interface layout). In the following section, we begin by introducing the notion of *persuasion*, drawing specifically on the ELM [18, 19] to explain the processes at work. We then link the issue of attitude change to the theoretical field of social representations. In particular, we discuss how social representations constitute a new avenue for improving the central processing of information. Finally, in the context of persuasive technology, we address interface design as a means of strengthening the sociocognitive relevance of the central elements of social representations, with the goal of shaping people’s attitudes so that, ultimately, they can become organ donors if they so wish.

### Initial concepts of persuasion and explanations for attitude change

*Persuasion* is broadly defined as the act of changing another person’s attitudes and behaviors [21]. For Miller, “any message that is intended to shape, reinforce, or change the responses of another, or others” can be regarded as persuasive communication (p. 11) [22]. Over the years, persuasion has been the subject of several different models [23–25], including learning [26, 27], cognitive response [28], dual [18, 29], and metacognitive models [30–32]. Among the latter, Salo and Karjaluoto [33] and Yoon [34] identified the ELM model [18, 19] as the most appropriate one for studying persuasive processes online.

The ELM model designed by Petty and Cacioppo [18] is based on Greenwald’s assumption that the cognitive responses produced by individuals after being exposed to an attempt at persuasion play a mediating role in the modification of attitudes [28]. First, individuals are asked to postpone any thoughts that came to mind during the exposure, and to relate the persuasive information to their previous attitude toward the topic in question. Then, in accordance with the self-validation model [29–31], they are asked to assign a valence (favorable, unfavorable or neutral) to the thoughts they had during the exposure. Greenwald [28] assumed that cognitively active individuals specifically link the information they process to their current feelings and beliefs.

The ELM includes motivational aspects and states that receivers processing a message can be placed on a continuum of elaboration or cognitive elaboration running from strong to weak.

In-depth processing is described as following a *central route* [18, 20]. Individuals mainly focus on the quality of the arguments, or at least engage in serious reflection about the message’s content. In this case, cognitive elaboration is considered strong. The resulting attitude is stable over time, insensitive to any counterpersuasive attempt, and predictive of subsequent behavior [35]. The message is embedded in the individual belief system. Superficial processing follows a *peripheral route*. The quality of argument is minimal [18, 20], and individuals proceed by heuristics.

The concept of *heuristics* relies more broadly on the heuristic-systematic model [29, 36], according to which two principles are involved in information processing: least effort [37] and (2) sufficiency [38]. These principles govern pre-established schemas in memory, allowing us to easily process information and effortlessly make decisions. When individuals follow the peripheral route, they focus on contextual clues, such as the credibility and expertise of the source of the message [20, 39], or even the pleasantness [40] or physical attractiveness of this source [41]. The resulting attitude is unstable over time, sensitive to counterpersuasive attempts, and not predictive of subsequent behavior [35].

Petty, Briñol, and Tormala [32] argued that individuals’ confidence in their own cognitive responses is key to persuasion. These authors therefore developed the self-validation model [30, 31], whereby the nature of the cognitive response is a necessary but not sufficient condition for attitude change. People must also cognitively validate their positive thoughts or doubt in the validity of their negative ones. Thought validation influences the effectiveness and stability of attitude change. Therefore, two kinds of cognitive variables play a role in attitude change: lower-level variables (cognitive responses) and high-level variables (metacognitions). *Metacognitions* are reflections about cognitions, and can be defined as *thoughts about thoughts* [42]. In the following section, we propose extending the notion of persuasion, and introduce the theory of social representations as a particularly useful means of influencing individuals’ ability and motivation to follow the central route when processing information.

### Contributions of social representations: Opportunities to follow the central route

*Social representations* reflect social actors’ ways of thinking and meaning making. According to Abric’s structural approach expressed within his *central core theory* ([43–45]; for reviews, see also [46–48]), the elements making up social representations are rooted in a dual system (central vs. peripheral). Central elements constitute the *core* of social representations. One of the main functions of this core is to preserve the stability of the representation over time. In particular, central elements constitute the most important part of the representation, fulfilling sense-making and sense-organizing functions. They are meaning markers and correspond to the group consensus [49]. Peripheral elements correspond to interindividual variability [44, 45]. They give additional meaning, by anchoring the representation in social reality and maintaining the context in which the representation emerged [45].

In the present study, we drew parallels between the notion of a core in social representations and the notion of schemas in Heider's theory of attributions [50]. According to this author, individuals make attributions in order to understand the social behaviors around them. More specifically, sociocognitive patterns operate in memory, allowing people to use causal attribution schemas and make predictions. This leads them to conceive of the world as regular, understandable, and explainable. The core of social representations is made up of stable cognitions. It provides access to knowledge about the world and shared reality [51]. As a result, central elements can be conceived of as a strong body of knowledge assigned to a particular object. The structural approach to social representations emphasizes the sociocognitive salience of the central elements in relation to the subject [52]. In an experimental study, Abric [53] found that central elements of social representations were recalled more than peripheral ones during a free recall task. Moreover, when central elements were absent from a list of words, participants systematically recalled them in a free recall task. Abric [53] therefore provided evidence of the sense-making and sense-organizing functions of the central elements. Just as Asch [54] argued that some central traits play a role in impression formation, so we postulated that the central core of social representations plays a similar role when it comes to lending meaning to an object.

In this respect, social representations may function as a network of beliefs about an object that are directly related to the corresponding attitudes [55]. With reference to the conceptions of Gestalt psychology, we can assume that the more salient a representational element is (i.e., belonging to the core), the more activated the object is in memory and the stronger the link with corresponding attitudes. These studies have opened up several new avenues of research, including the interconnection between social representations and so-called *binding communication* [56, 57]. It have been shown that involving central elements of social representations, rather than peripheral ones, in the preparatory act ultimately leads to greater commitment effects at both cognitive and behavioral levels (for reviews, see [58, 59]).

Some evidence in favor of these mechanisms has come from Zbinden et al. [57]. These authors found that participants who were exposed to central elements of social representations of environmental protection were more likely to achieve the query target (i.e., devote about 15 minutes of their break time to advising spectators at a sporting event on how to sort waste) than those who were not exposed to any elements. In addition, these participants expressed a stronger intention to carry out recycling themselves.

In the present study, we hypothesized that including the central elements of social representations in a message strengthens its persuasive impact, particularly by encouraging people to follow the central route in processing information. Furthermore, with regard to persuasive technology, we reasoned that an appropriate interface design can improve the persuasive reach of a message. We therefore used interface design to improve the salience of the central elements.

### Interface layout: Opportunities to improve the persuasive impact of information displayed

Interface design, in terms of the location of the information on the screen, as well as the choice of color and contrast, can serve to highlight the content of the message and, in our study, the central elements of particular social representations. We postulated that persuasive technology can be used to meet users’ needs and guide decision-making [60] through a process of nudging. Thaler and Sunstein [61] introduced the concept of *nudging* to suggest that our knowledge about biases in decision-making can be used to support individuals in making good decisions. A nudge is “any aspect of the choice architecture that alters people’s behavior in a predictable way without forbidding any option or significantly changing their economic incentive” (p. 6). Nudging has been applied in human-computer interaction, as well as in other domains, including marketing, social marketing, and public policy (e.g., [62]), with the overall aim of facilitating the processing of important data [63] and providing individuals with a shortcut to action and behavior change [64].

Bearing in mind Fogg’s conception of computers as tools, interactions, and social actors [10], designers rely on *ergonomics* (or *human factors*) to devise persuasive technology, and thus on a deep understanding of the interactions between humans and other elements of a system [65]. One of the main objectives nowadays is to focus on users’ feelings and emotions during the human-computer interaction [66–68] and to look for a good *user experience* (UX) [69]. In our study, we explored the effects of two specific features of interface design concerning how a message is displayed to users: (1) *location of the information* and (2) *background characteristics* (i.e., color and contrast).

**Fig 1:**
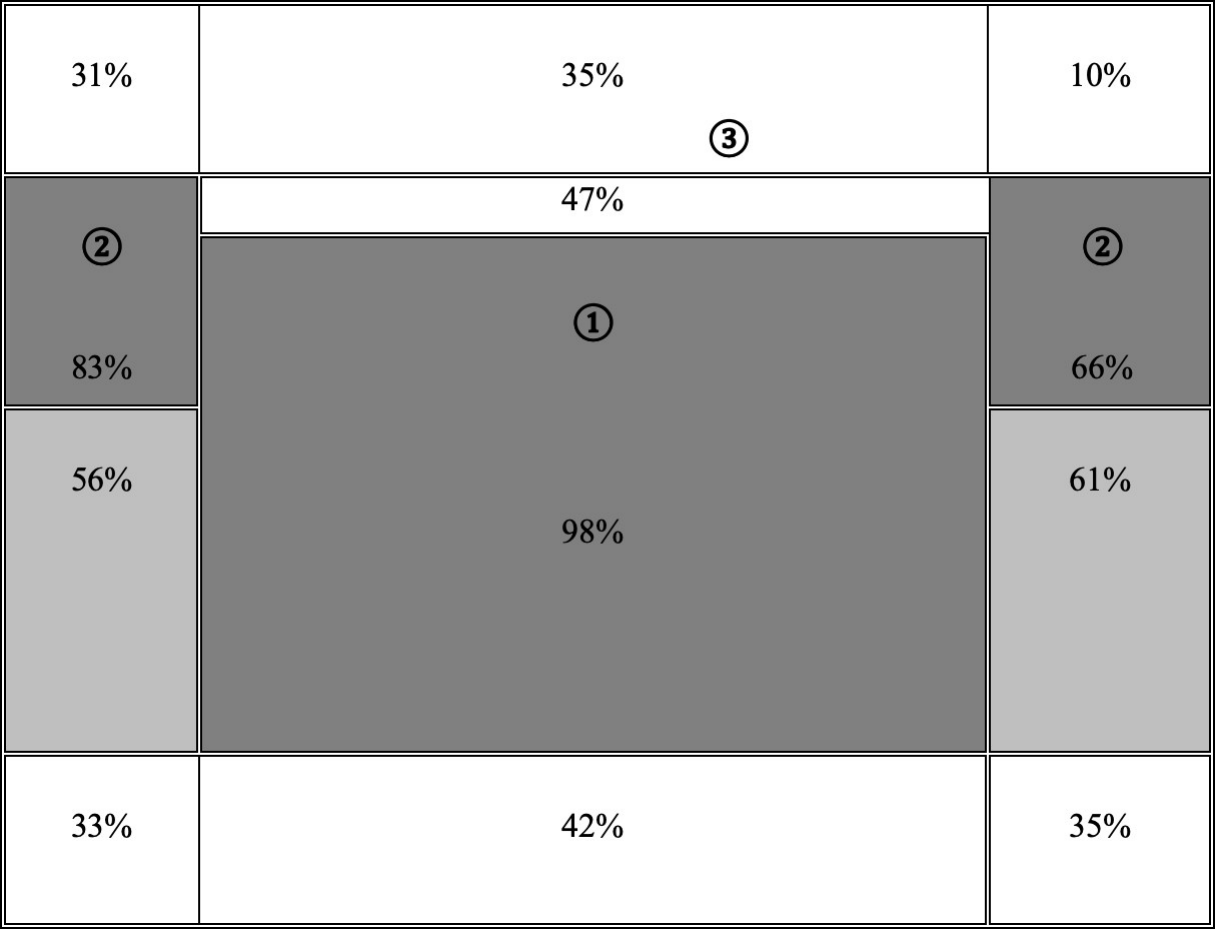
Order and percentage of fixations by internet users on a web page. Schema taken from Nogier, Bouillot, and Leclerc [71], p. 33. The percentage corresponds to the proportion of internet users who fixate the relevant area at least once.

In terms of location, there are several constants in the way people first look at a screen. According to Nogier, Bouillot, and Leclerc [70], they fixate the middle of the screen first, and only then does their gaze shift to the left and to the right (Fig 1). It therefore seems better to place a message at the top of the screen in the middle to maximize its visibility (see Areas 1, 2 and 3 in Fig 1). Areas on the sides that receive only 33%, 31% and 10% of views seem the least appropriate places for displaying information. Therefore, in addition to using the status of social representation elements (i.e., central or peripheral), we argue that displaying information in locations that people spontaneously view first can force them to pay direct attention to the content [71].

In terms of background characteristics, early research showed that short-wavelength colors, such as blue and green, are more pleasant and are preferred to long-wavelength colors, such as red and yellow [72, 73]. More specifically, long-wavelength colors trigger anxiety, whereas short-wavelength colors generate pleasure [74, 75]. In this vein, blue is usually associated with calmness and trust [76, 77] and tends to be universally valued [78, 79]. In addition, in the information context, a blue website, as opposed to one in another color, appears to favor not only users’ satisfaction but also their efficiency and memorization of the information [80]. Moreover, in the marketing context, website design and color can be used to promote the formation of positive opinions [81], as well as purchase intentions [82].

Designing interfaces to improve persuasiveness also involves focusing on contrast and luminance, in addition to color. In particular, researchers recommend maintaining a high contrast between the text and the background in order to enhance readability [83, 84], which appears to be directly linked to pleasantness [85]. Moreover, readability can be more dependent on luminance than on color [86]. Greater contrasts between text and background color lead to faster searching [87, 88]. More specifically, black on white is more familiar to users [88, 89], helps reduce visual fatigue [90], and may be the best combination in terms of optimum readability, reading speed, comprehension, and preference [85]. Therefore, by improving readability, a high-level contrast may allow information (e.g., central elements of social representations) to be highlighted.

## Objectives

The first goal of the present study was to explore whether the sociocognitive salience of the central elements of social representations can be used to achieve persuasive outcomes through the use of persuasive technology, specifically in the context of organ donation. The second goal was to explore how the display format, in terms of the location of the message and the background, can make the central elements of social representations more relevant. In particular, we wished to identify the most relevant characteristics (color vs. contrast) in terms of persuasiveness.

## Overview

After conducting a preliminary study to determine the content and structure of social representations of organ donation, we conducted our main study in five stages. First, we collected each participant’s prior attitude toward organ donation. Second, each participant was seated in front of a computer screen and exposed to elements of social representations of organ donation, printed in black. This stage involved three between-participants variables: (1) status of social representation elements (central vs. peripheral), (2) location of social representation elements on the screen (middle vs. side), and (3) background color (blue vs. white). During this stage, an eye-tracking device recorded participants’ eye movements. Third, participants were each asked to complete a questionnaire that probed, for example, their attitude and intention toward organ donation. Fourth, we assessed participants’ behavior, by asking them to place a sticker featuring the words “I am an organ donor” on their mobile phone. Fifth, we checked their behavioral commitment a fortnight after the experiment, by sending each of them an e-mail asking them to publish a readymade poster promoting organ donation on Facebook.

## Hypotheses

First, regarding the persuasive argument, we expected participants to report the message content *more accurately* when it concerned central rather than peripheral elements, in line with Abric’s demonstration of better retention when individuals are exposed to central rather than peripheral elements of the social representations [53].

Second, based on the assumption that the central elements of social representations have an epistemic function [91] and fulfil human needs for knowledge and sharing [51], we expected *reported satisfaction* to be higher when participants were exposed to central rather than peripheral elements of the social representations.

Third, because of the greater salience of the central elements of social representations [52, 53], we predicted that when these elements (vs. the peripheral ones) were displayed, participants would follow the *central route* for processing information rather than the peripheral one [21, 35]. Consequently, in accordance with the self-validation model [30–32], we expected participants to report *more favorable thoughts and more validation* of their thoughts when they were exposed to central rather than peripheral elements of the social representations. We also expected participants to exhibit stronger, more favorable and *more certain attitudes* toward organ donation when they had been exposed to central rather than peripheral elements of the social representations. Finally, we expected participants to express *stronger intentions* to become organ donors. We also expected them to be *more likely to place the organ donor sticker* on their phone and *publish the poster* on social media if they had been exposed to central rather than peripheral elements of the social representations.

In terms of interface layout, we first expected participants to focus more (in terms of *number of fixations*, *fixation time*, and *number of lookbacks*) on the representational elements when these were displayed in the middle of the screen rather than on the side. Second, by displaying elements in the areas of the screen that are spontaneously viewed first [70], we hoped to meet participants’ needs [66]. More specifically, with reference to Cornish et al. [63], we hoped to facilitate the processing of important data and reduce cognitive demand through the design of the interface. Consequently, we expected participants to express *greater satisfaction* after being exposed to elements placed in the middle of the screen rather than on the side, especially when these were central rather than peripheral elements of the social representations. Finally, given the strong positive correlation between satisfaction and persuasion [92], we expected participants to report *more favorable attitudes*, *intentions* and *behaviors* toward organ donation when they were exposed to elements displayed in the middle of the screen rather than on the side, especially when it came to central elements of the social representations.

In terms of background characteristics, we first expected participants to express greater satisfaction when they were exposed to a blue (rather than a white) background, the former being universally appreciated [78, 79]. Given the greater readability of black text against a white background [84], we further assumed that focusing on optimum contrast (e.g., black on white) would enhance the salience of the central elements of the social representations. Therefore, we ultimately expected that focusing on optimum contrast rather than color would be more effective in terms of persuasiveness. In particular, participants should be able to report more favorable *attitudes*, *intentions* and *behaviors* toward organ donation when central rather than peripheral elements of the social representations were displayed against a white background than a blue one.

In terms of interaction effects between content and layout, we assumed that displaying information in the middle of the screen rather than on the side, and against a white background rather than a blue one, would allow this information to be highlighted and, ultimately, be more persuasive. More specifically, we expected participants to report stronger, more favorable and more certain attitudes, more favorable intentions, and more favorable behaviors toward organ donation, after being exposed to central rather than peripheral elements of the social representations, all the more so when these central elements were displayed in the middle of the screen against a white background.

We of course expected to observe several within-participants correlations among the different measures. For instance, in line with the ELM [18, 19] and the self-validation model [30–32], we excepted to find significant positive correlations between attitude and intention and thought validation. With reference to Nanou et al. [92], we also expected satisfaction to be predictive of attitude and intention, after controlling for prior attitude toward organ donation.

## Preliminary study to assess social representations of organ donation

### Materials and methods

#### Participants

For both the preliminary and the main study, participants were directly recruited on a university campus. They were all French first-year psychology undergraduates.

A total of 121 participants took part in the preliminary study. Of these, 91 participated in the collection of the content of the social representations (63 women; *M*_age_ = 19.04 years, *SD* = 1.58), and 30 participated in the identification of the structure of the social representations (21 women; *M*_age_ = 19.87 years, *SD* = 2.03). They were all guaranteed anonymity, and were told they could withdraw from the study at any time. This preliminary study was approved by the ethics committee of Aix Marseille University.

#### Procedure

To collect the content of the social representations, 91 students completed a paper questionnaire. In accordance with the literature [93–95] (for a review, see [48]), the questionnaire began with a *hierarchical evocations* verbal association task. First, participants were asked to associate the four words or expressions that came to mind when we said "organ donation". Second, they had to rank these words or expressions from 1 (*the most important in relation to the object*) to 4 (*the least important in relation to the object*). Third, participants had to produce one sentence explaining the meaning they gave to these words or expressions in relation to organ donation. This step (*semantic contextualization*) [96, 97] allowed us to perform a thematic content analysis [98]. Our goal was to gather evocations in a limited number of categories. Once we had completed the thematic analysis, we ran a prototypical analysis, crossing the frequency of occurrence of the words or expressions with their ranking [93]. This analysis allowed us to highlight specific elements of the social representations. We hypothesized that the themes that were most frequently cited and rated as the most important formed the central core of the social representations [48]. Other elements that were either frequently or infrequently cited, but given a lower ranking, were assumed to have peripheral status. We submitted these hypotheses to a test of centrality [48, 93].

To identify the structure of the social representations, 30 different students responded to a test of context independence (TCI; [99]; for a review, see [48]). More specifically, the items in this test included the 12 most salient themes in terms of frequency and importance, according to the prototypical analysis we had carried out. Items were all formulated as follows: "In your opinion, is organ donation always, in every case, an act of generosity?" or "In your opinion, does organ donation always, in every case, save lives?" For each item, participants had to indicate their answer on a 4-point Likert scale ranging from 1 (*Definitely no*) to 4 (*Definitely yes*).

In accordance with the TCI methodology and with previous studies [48, 49, 99, 100], we calculated a percentage of centrality for each element. This percentage was then compared with a decisional threshold, and all items with a percentage of centrality greater or equal to this threshold were deemed to be central, while all items with a percentage of centrality below the threshold were deemed to be peripheral elements (see S1 Appendix for details of the procedure).

### Results

The results of the TCI are reported in Table 1.

**Table 1:** Results of the TCI for social representations of organ donation.

Social representations of organ donation	
Items	TCI
**Help**	96.7^a^
**Save lives**	93.3 ^a^
**Involves body**	93.3 ^a^
**Good deed**	90 ^a^
**Honorable**	90 ^a^
**Necessary**	90 ^a^
**Donation**	86.7 ^a^
**Transmission**	83.3 ^a^
**Generosity**	70
**Illness**	60
**Evidence**	50
**Death**	36.7

^a^ Elements identified as central based on the values in the Kolmogorov-Smirnov table with a TCI centrality threshold (*N* = 30) of 76%.

In the main study, we treated *help*, *save lives* and *good deed* as the central elements of the social representations of organ donation, and *generosity*, *illness* and *evidence* as the peripheral ones (see Table 1).

## Main study

### Materials and methods

#### Participants

Participants were 240 undergraduates, who received a course credit for taking part (218 women; *M*_age_ = 19.27 years, *SD* = 1.68). None of them had been informed of the purpose of the experiment. All of them provided their written informed consent before participating and were guaranteed confidentiality and the opportunity to withdraw from the study at any time. This experiment was approved by the ethics committee of Aix Marseille University.

#### Procedure

The 240 participants were randomly assigned to one of the eight conditions of the experiment (30 per condition).

In order to include a control variable, we recorded participants’ prior attitude toward organ donation. To this end, we asked participants to say how much they supported organ donation on a scale ranging from 0 (*Not at all*) to 10 (*Quite favorable*). Next, each participant was seated in front of a computer screen and exposed to two successive sequences of stimuli. Instead of a fixation cross, the first slide served as a prime, featuring the sentence "Organ donation is…". This sentence was displayed in the middle of the screen for 5 seconds, during which no measurement was recorded. The second slide lasted 7 seconds and introduced participants to three words printed in black. There were three independent variables (IVs), as the words referred to either central or peripheral elements of the social representations of organ donation (status IV), and were displayed either in the middle or on the side of the screen (location IV), against a white or a blue background (background IV).

Eye movements were recorded with a Tobii Studio® eye-tracking device. A fixation was defined as lasting longer than 50 ms. Signals were sampled and stored at a rate of 60 Hz. Recording was binocular, measuring both right and left eye movements, and calibration was performed before each participant started the experiment. In persuasive contexts, eye-tracking devices can be used to provide realtime information about visual attention allocation [100]. We reasoned that accessing attentional processes would provide us with a better understanding of how individuals behave in the presence of persuasive technology. There were 13 dependent variables (DVs). For a start, we measured three types of eye movements. First, the *total number of fixations* (DV 1) was defined as the total number of fixations per participant on the areas of interest (AOIs). Three AOIs (one for each word) were drawn on the slide where the persuasive message was displayed, regardless of experimental condition. A fixation on a specific displayed element was assumed to reflect the importance of that element for the participant [101–103]. We then summed the numbers of fixations for each of the three AOIs. Second, the *total fixation time* (DV 2) was defined as the total amount of time spent by each participant fixating the AOIs. A longer fixation was interpreted as indicating that this specific displayed element was more engaging for the participant [101]. We then summed the fixation time for each of the three AOIs. Third, the *total number of lookbacks* (DV 3) was defined as the number of new fixations made by the participant on the AOIs, after having already fixated it once. For each AOI, we calculated the number of lookbacks by taking the number of fixations made on that AOI, minus one. We then summed the numbers of lookbacks for each of the three AOIs. We expected these lookbacks to provide us with additional information about the participants’ level of interest in each interface design [101, 102]. Thereafter, to clarify their intentions, we administered self-report measurements to participants.

Participants were asked to answer an online questionnaire displayed on the same computer (see S2 Appendix for the items of this questionnaire). The questionnaire began with a satisfaction measurement: “How satisfied are you with the previous slide on a scale from 0 (*Not satisfied at all*) to 10 (*Quite satisfied*)?” (DV 4). We then carried out attitude measurements (DVs 5-8). First, we collected participants’ explicit attitudes with the following question: “At this time, on a scale from 0 (*Not favorable at all*) to 10 (*Quite favorable*), how would you rate your support for organ donation?” Second, we collected attitude strength on a scale ranging from 0 (*Not sure at all* or *Not important at all*) to 10 (*Quite sure* or *Quite important*): “How sure are you of the answer you gave to the previous question?” and “How important would you say that organ donation is to you?” We also assessed participants’ behavioral intentions: “At this time, on a scale from 0 (*I have no intention at all*) to 10 (*I fully intend*), how much would you estimate your intention to declare yourself an organ donor?” We also collected participants’ thoughts and their validation of these thoughts (DVs 9 & 10). First, participants had to write down all the thoughts that came to mind when they saw the slide. Second, they had to assign a valence to each of these thoughts by entering a zero if they found it neutral, a plus sign if they found it favorable, and a minus sign if they found it unfavorable. We coded these valences +1 for *favorable* thoughts, -1 for *unfavorable* thoughts, and 0 for *neutral* thoughts, and computed a main score. Finally, we accessed participants’ validation of their thoughts by asking them: “Now, could you indicate whether you were confident when you had these thoughts? Please rate your answer on a scale from 0 (*Not confident at all*) to 10 (*Quite confident*)”. The last part of the questionnaire featured free recall of the words displayed in the second slide (DV 11). We also collected sociodemographic data including sex, age, and experience-related data (“Have you received at least one organ transplant?”, and “Do you know people around you who have received an organ transplant or who are waiting for a donor?”). Once the questionnaire had been completed, we suggested to each participant that they take a sticker saying "I am an organ donor" (DV 12). We asked them to put it on their cellphone in order to later publicly assert their positioning to others. If participants did not have a cellphone, they could put the sticker on their laptop. Each participant was free to accept or reject this behavioral request. We also checked participants’ behavioral commitment. A fortnight after the experiment, participants were contacted by email and asked to publish a poster promoting organ donation on Facebook (DV 13). Out of the 240 participants in our experiment, 178 had an identifiable Facebook account. The poster has been designed by the French Biomedicine Agency. Publishing the poster in a public space might increase commitment [104]. This allowed us to check that this behavior had actually been performed, simply by typing each participant’s profile name. More particularly, we did not have to be one of the participants' *friends* to carry out this check.

#### Data processing and analysis

We did not expect prior attitude to interact with interface layout, as location and color are features that are fully independent of the evaluation of the object (here, organ donation). However, as stated earlier (see also Rouquette [105]), attitudes are particularly rooted in social representations and we therefore took the effect of prior attitude into account whenever the status IV was involved.

For the quantitative data, we performed analyses of variance (ANOVAs), Pearson correlations and regressions. For the dichotomous qualitative data (i.e., behavioral variables), we ran chi-square tests.

### Results

Of all the possible interactions, only two were found to be significant: the three-way Status x Location x Background interaction had a significant effect on attitude certainty, *F*(1, 239) = 7.18, *p* = .008, η^2^ = .03, and the two-way Status x Location interaction had a significant effect on number of fixations, *F*(1, 238) = 4.79, *p* = .03, η^2^ = .02. No other interaction yielded a *p* value below the consensual threshold of *p* < .05. The Results section therefore overwhelmingly contains analyses of main effects, given that (1) interaction effects were scarce and related to specific DVs, and (2) when the IVs were combined, instead of yielding divergent results, they shaped the DVs in a similar fashion whatever the interaction. Therefore, we consider that essentially describing the main effects does not reduce the overall picture of either our data or their real-world significance. For the sake of clarity, the following section is ordered according to our hypotheses.

#### Main effects of content of the persuasive argument

A one-way ANOVA revealed several significant main effects of the structural status of social representations (see Table 2).

**Table 2:** Effects of structural status of social representations (summary of ANOVA results).

Dependent variable	Terms of independent variable	*M*	*SD*	95% CI	*F*	*p*	η_p_^2^
**Free recall**	Central	1.60	.79	[1.45, 1.74]	10.60	.001	.043
	Peripheral	1.93	.83	[1.79, 2.08]			
**Satisfaction**	Central	7.14	2.36	[6.68, 7.48]	9.90	.002	.040
	Peripheral	6.12	2.27	[5.77, 6.58]			
**Thoughts (valence)**	Central	2.11	1.48	[1.82, 2.32]	15.60	< .001	.063
	Peripheral	1.31	1.44	[1.10, 1.60]			
**Thought validation**	Central	8.12	1.98	[7.71, 8.43]	8.22	.005	.034
	Peripheral	7.28	2.14	[6.97, 7.69]			
**Attitude**	Central	8.49	1.95	[8.18, 8.49]	.062	.804	.000
	Peripheral	8.20	2.00	[8.21, 8.51]			
**Attitude certainty**	Central	8.55	2.15	[8.12, 8.80]	.041	.840	.000
	Peripheral	8.32	2.16	[8.07, 8.75]			
**Importance of attitude**	Central	8.08	2.20	[7.64, 8.29]	2.07	.152	.009
	Peripheral	7.52	2.23	[7.31, 7.96]			
**Intention**	Central	7.57	2.96	[7.00, 7.74]	2.37	.125	.010
	Peripheral	6.77	3.03	[6.60, 7.33]			

As shown in Table 2, results indicated a main effect of structural status of the social representations on free recall, *F*(1, 238) = 10.60, *p* < .05, η_p_^2^ = .043. Contrary to our assumptions, participants recalled more words on average when they were exposed to peripheral, rather than central, elements of the social representations. Second, results indicated a main effect of structural status on satisfaction. On average, participants reported greater satisfaction when they were exposed to central rather than peripheral elements. Third, results showed a significant effect of structural status on the valence of thoughts. On average, participants produced more favorable thoughts when they were exposed to central elements of the social representations rather than peripheral ones. Here, data were missing for two participants, as they did not respond to this item of the questionnaire. These two participants did not assign valence to their thoughts by entering a zero, a plus sign or a minus sign. Analyses also revealed that participants expressed greater confidence in their thoughts in the central status condition than in the peripheral one. We found no significant effect of structural status on attitude, attitude strength, or intention. However, a significant effect was found on the first behavior, as revealed by a chi-square test, χ^2^(1, *N* = 238) = 7.14, *p* < .01, ϕ = .173. Participants were more willing to declare themselves to be organ donors by putting the sticker on their phone after being exposed to central rather than peripheral elements of the social representations. More specifically, 105 participants agreed to take the sticker in the central status condition, compared with only 89 in the peripheral status condition. Nevertheless, we did not find this relationship with respect to the second target behavior (i.e., publishing the poster), χ^2^(1, *N* = 178) = .295, *ns*. Out of the 178 participants who were on Facebook, five exhibited the target behavior in the central status condition, and six in the peripheral status condition.

#### Main effects of interface layout

First, a one-way ANOVA revealed several significant main effects of location of the persuasive argument on the screen (see Table 3).

**Table 3:** Effects of location of the persuasive argument on the screen (summary of ANOVA results).

Dependent variable	Terms of the independent variable	*M*	*SD*	95% CI	*F*	*p*	η_p_^2^
Number of fixations	In the middle	56.65	14.66	[54.08, 59.23]	10.13	.002	.041
	On the side	50.78	13.73	[48.22, 53.37]			
Fixation time (ms)	In the middle	4.26	1.08	[4.07, 4.45]	31.96	< .001	.119
	On the side	3.48	1.05	[3.29, 3.67]			
Number of lookbacks	In the middle	5.65	2.32	[5.26, 6.04]	17.08	< .001	.068
	On the side	4.50	1.93	[4.12, 4.88]			
Satisfaction	In the middle	6.93	2.32	[6.51, 7.35]	3.93	.049	.016
	On the side	6.33	2.39	[5.90, 6.76]			
Attitude	In the middle	8.52	1.80	[8.20, 8.85]	1.86	.174	.008
	On the side	8.17	2.13	[8.09, 8.60]			
Difference in attitudes (i.e., general attitude toward organ donation, collected before and after the experiment)	In the middle	.076	1.00	[-.085, .236]	.896	.345	.004
	On the side	-.033	.76	[-.192, .126]			
Attitude certainty	In the middle	8.49	2.16	[8.10, 8.88]	.148	.700	.001
	On the side	8.38	2.15	[7.99, 8.77]			
Importance of attitude	In the middle	7.61	2.24	[7.21, 8.02]	1.66	.199	.007
	On the side	7.98	2.21	[7.59, 8.38]			
Intention	In the middle	7.30	2.79	[6.80, 7.81]	.478	.490	.002
	On the side	7.03	3.23	[6.45, 7.61]			

The ANOVA results (Table 3) showed a main effect of location on number of fixations. On average, individuals made more fixations on elements when these were displayed in the middle of the screen rather than on the side. We also found a main effect of location on fixation time. On average, elements of the social representations were fixated for longer when they were displayed in the middle of the screen rather than on the side. We found a main effect of location on number of lookbacks. Individuals generally made more lookbacks when elements of social representations were displayed in the middle of the screen rather than on the side. There was also a main effect of location on satisfaction. On average, individuals expressed greater satisfaction with the interface when elements of the social representations were located in the middle rather than on the side. We found no significant effect of location on attitude, attitude strength, or intention. Chi-square tests were performed to explore the effects of location on the first and second target behaviors. Results of these tests were both nonsignificant: χ^2^(1, *N* = 238) = .367, *ns*, and, χ^2^(1, *N* = 178) = .074, *ns*. Out of 238 participants, 98 agreed to take the sticker in the middle condition, and 96 in the side condition. Of the 178 participants who were on Facebook, five agreed to publish the poster in the middle condition, and six in the side condition.

A one-way ANOVA revealed several significant main effects of background (see Table 4).

**Table 4:** Effects of color and contrast background characteristics (summary of ANOVA results).

Dependent variable	Terms of the independent variable	*M*	*SD*	95% CI	*F*	*p*	η_p_^2^
Satisfaction	White	6.65	2.33	[6.21, 7.05]	.010	.919	.000
	Blue	6.61	2.41	[6.20, 7.00]			
Attitude	White	8.59	1.70	[8.26,8.89]	3.66	.057	.015
	Blue	8.10	2.20	[7.67, 8.48]			
Difference in attitudes	White	.107	.668	[-.265, .050]	5.17	.024	.021
	Blue	.151	1.05	[-.008, .310]			
Attitude certainty	White	8.41	2.26	[8.02, 8.79]	.042	.837	.000
	Blue	8.46	2.05	[8.07, 8.85]			
Importance of attitude	White	7.79	2.38	[7.35, 8.19]	.002	.963	.000
	Blue	7.80	2.07	[7.44, 8.18]			
Intention	White	7.22	2.94	[6.71, 7.74]	.085	.771	.000
	Blue	7.11	3.10	[6.56, 7.66]			

Contrary to our initial assumptions, the ANOVA (Table 4) failed to show any significant effect of background on either satisfaction, attitude strength, or intention. Results only revealed a marginal effect on attitude, indicating that people may report a more favorable attitude toward organ donation when exposed to a white rather than a blue background. This was consistent with our initial assumption. We found a significant effect of background on difference in attitudes. However, the direction of this effect was not consistent with our initial assumption, for on average, people changed their attitude more when the persuasive message was displayed against a blue rather than a white background. As the color blue tends to be universally liked, this effect may be in line with Nanou, Lekakos, and Fouskas [92], who found a strong positive correlation between satisfaction and persuasion. However, we were not able to confirm our assumption about satisfaction. Furthermore, the results of chi-square tests performed to explore effects of background characteristics on the first and the second target behaviors were nonsignificant, both for the first behavior, χ^2^(1, *N* = 238) = .015, *ns*, and for the second behavior, χ^2^(1, *N* = 178) = 1.02, *ns*. Out of 238 participants, 99 agreed to take the sticker in the white condition, and 95 in the blue condition. Of the178 participants who were on Facebook, four agreed to publish the poster in the white condition, and seven in the blue condition.

#### Three-way interaction effects between content of the persuasive argument and interface layout

A three-way Status x Location x Background ANOVA revealed a significant effect on attitude certainty, but not on any of the other DVs. The most relevant results relative to our assumptions are reported in Table 5.

**Table 5:** Summary of three-way Status x Location x Background interaction effects.

**Dependent variable**	**Terms of the independent variable**			*M*	*SE*	*F*	*p*	η_p_^2^
	**Status**	**Location**	**Background**					
Attitude	Central	In the middle	White	9.40	1.00	2.19	.140	.009
	Peripheral	On the side	Blue	8.10	2.25			
Attitude certainty	Central	In the middle	White	9.30	1.09	7.18	.008	.030
	Peripheral	On the side	Blue	8.37	1.99			
Importance of attitude	Central	In the middle	White	8.57	2.08	3.17	.076	.014
	Peripheral	On the side	Blue	7.97	2.20			
Intention	Central	In the middle	White	8.33	2.14	.001	.980	.000
	Peripheral	On the side	Blue	7.13	3.14			

**Fig 2:**
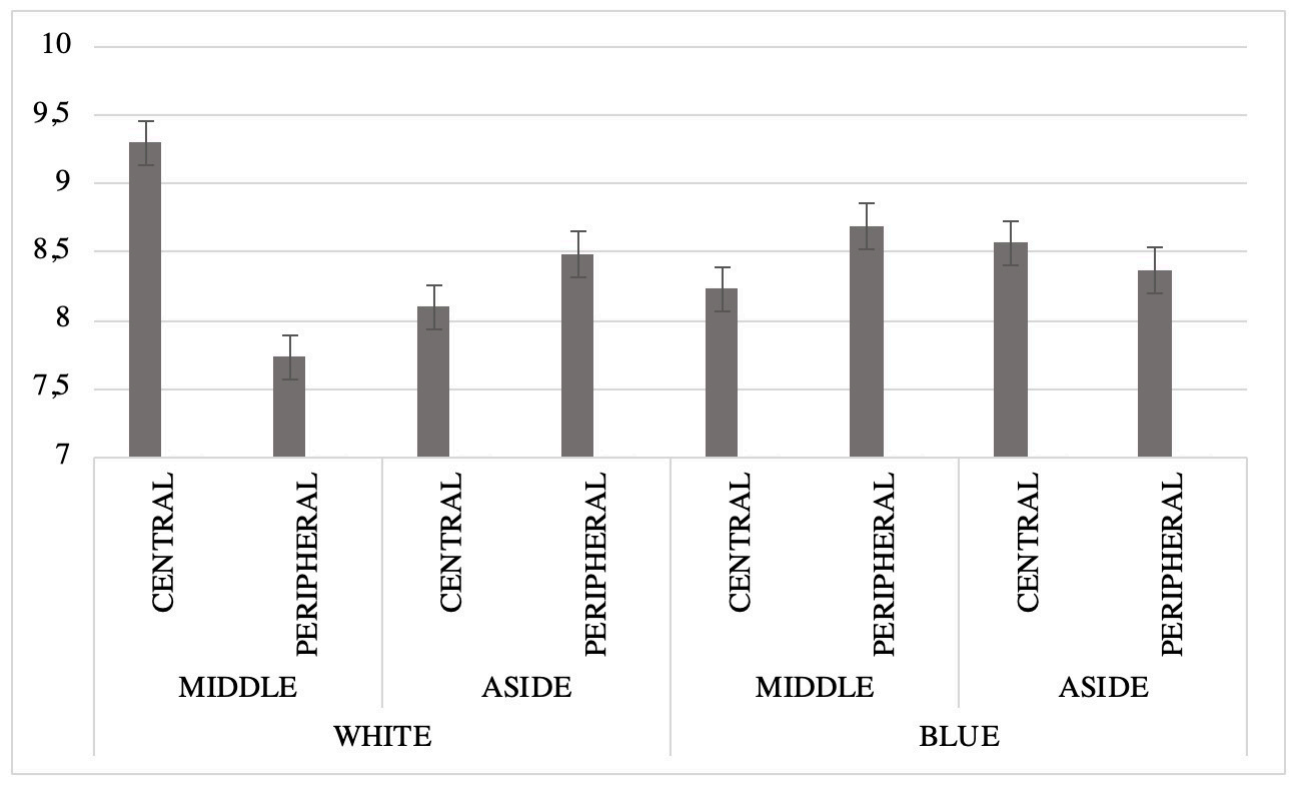
Results of ANOVA performed on status, location and background, regarding effects on certainty of attitudes.

As shown in Table 5, we found a significant Status x Location x Background interaction effect on attitude certainty (see Fig 2). On average, the most certain attitudes appeared when central elements were displayed in the middle of the screen against a white background. In all the other conditions, central elements were treated with less certainty, while peripheral elements were either treated with less certainty or else did not differ from central elements on certainty.

We found no significant Status x Location x Background interaction effect on attitude, importance of attitude, or intention. Chi-square tests were performed to explore the effects of the interaction between the content of the persuasive argument and interface layout on the first and second target behaviors. The results for the first behavior are reported in Table 6.

**Table 6:** Effects of Status x Location x Background interaction on first target behavior (summary of chi-square tests).

Dependent variable		Terms of the independent variable			χ^2^	*df*	*p*	ϕ
(agreeing to take the sticker)								
Yes	No	Status	Location	Background				
29	1	Central	In the middle	White	6.41	1	.011	.327
22	8	Peripheral						
26	4	Central	On the side	White	2.24	1	.134	.192
22	9	Peripheral						
99	22	Total	Total	White	7.76	1	.005	.253
26	3	Central	In the middle	Blue	2.81	1	.094	.220
21	8	Peripheral						
24	6	Central	On the side	Blue	.074	1	.786	.111
24	5	Peripheral						
95	22	Total	Total	Blue	.982	1	.322	.092

As shown in Table 6, structural status had an effect on the first target behavior when the central elements were shown against a white background but not a blue one, and when they were displayed in the middle but not on the side. Results of chi-square tests were both nonsignificant in the blue condition. We found no significant effect of the interaction between the content of the persuasive argument and the interface layout on the second target behavior.

#### Persuasive process

We carried out Pearson correlation coefficients between the eye-tracking data, and attitude and intention toward organ donation. We only found one significant correlation, between fixation time and intention, *r*(236) = .16, *p* < .05. Thus, as fixation time increased, the intention to declare oneself an organ donor increased too.

Additionally, we checked the postulates of the self-validation model [30–32]. Pearson correlation coefficients were performed between thought validation (favorable, unfavorable, or neutral) and attitude and intention toward organ donation. First, thought validation was correlated with general attitude, *r*(240) = .31, *p* < .001. Second, it was correlated with attitude strength, *r*(240) = .37, *p* < .001, and certainty, *r*(240) = .41, *p <* .001. Third, it was correlated with intention, *r*(240) = .28, *p* < .001.

Furthermore, in line with the theory of reasoned action [106], hierarchical multiple regressions predicted intention toward organ donation from attitude. More specifically, intention was predicted by attitude, *F*(2, 239) = 144.84, *p* < .001, *R*^2^ = .55, certainty, *F*(2, 239) = 153.74, *p* < .001, *R*^2^ = .57, and importance, *F*(2, 239) = 185.44, *p* < .001, *R*^2^ = .61.

Finally, in line with Nanou et al. [92], hierarchical multiple regressions predicted persuasion effects from satisfaction. We found that satisfaction predicted attitude, *F*(2, 239) = 572.37, *p* < .001, *R*^2^ = .83, difference in attitudes (i.e., collected before and after the experiment), *F*(2, 239) = 21. 18, *p* < .001, *R*^2^ = .15, and intention, *F*(2, 239) = 142.22, *p* = .001, *R*^2^ = .55.

## Discussion

First of all, the present study supported the expected relevance of associating the theory of social representations with experimental designs exploring persuasive technology. More specifically, our results showed that participants exposed to central elements of the social representations of organ donation, rather than peripheral ones, ultimately reported more favorable attitudes and intentions toward organ donation. Additionally, more individuals exposed to central rather than peripheral elements of the social representations produced the first target behavior (i.e., declaring themselves to be organ donors, by taking the sticker). At the same time, we found that the effects of attitudes and behaviors could be explained by the predictions of (1) the ELM [18, 19], and (2) the self-validation model [30–32]. In particular, this study demonstrated that individuals follow the central route of information processing when they are exposed to the central elements of social representations. On average, participants exposed to central rather than peripheral elements reported more favorable thoughts, and greater confidence in these thoughts. Then, in line with the self-validation model, we confirmed that thought validation is positively correlated with attitude and intention toward organ donation. Lastly, in line with the theory of reasoned action [106], we found that intention was predicted by attitude.

This study also highlighted the value of taking the effects of interface characteristics into account when designing persuasive technology. In particular, the effect of status on attitude certainty did not occur independently of the interface layout. The most certain attitudes appeared when the central elements were displayed in the middle of the screen against a white background.

First, we argue that displaying the message in the middle of the screen increased the relevance of its content, namely the salience of the central elements of the social representations. Second, we argue that the marked contrast of the black text against the white background improved readability, compared with the same text displayed against a blue background. Moreover, while blue is perceived of as satisfying or pleasant (*peaceful* or *calming*), according to the literature [74, 75, 80], the feelings conveyed by this color may not be appropriate for a societal issue such as organ donation and the need to make important and binding decisions about it. When we considered this Status x Location x Background interaction, we observed that blue led to the smallest differences in terms of attitude certainty, as if participants were less influenced by the elements’ other characteristics (i.e., status and location).

Finally, we were unable to confirm behavioral commitment in the present study. There are several possible explanations for this. First, according to the associative interference model, which predicts that any attitude change decreases over time [20], it is possible that participants quickly forgot the message content after the experiment. Second, when a new attitude is formed, the old one is not necessarily rejected [107]. New and old attitudes can coexist, with one being expressed at the conscious level (the new one), and the other at the implicit level (the old one). When people have to undergo a change, their implicit attitudes may influence their judgments and behaviors, if they are not willing or able to engage in the laborious process of retrieving their new attitude in memory. Third, the lack of commitment we observed may have been due to the experimental design. All the participants in this experiment were students who received a course credit for their participation. According to the theory of self-perception [108, 109], once the experiment was over, participants may have analyzed their attitude and the first behavior in terms of external conditions, but subsequently linked it to internal causes (e.g., “I did it to get a credit, so I did not do it for my own reasons. Therefore, I am not motivated to perform the second target behavior”).

### Limitations

The first limitation concerned the student sample. Although students are an important target group for organ donation campaigns, future research should look at whether the results of this study can be generalized to broader populations. The second limitation concerns the interconnection between attitudes and the social contexts in which these attitudes are expressed [110]. For example, the expression of attitudes can regulate social interactions. In our experiment, the processes of persuasion, and in particular the expression of favorable attitudes and behaviors toward organ donation, may have been subject to a social desirability bias. It is therefore necessary to study how the attitudes that individuals express can be influenced by self-patterns (e.g., goals, motivations, behaviors, affects) during information processing. Furthermore, it would be relevant to study how self-patterns can play a specific role in social perception, comparison, and interactions.

### Conclusion and implications

Our study makes several contributions to both research and practice.

From a theoretical perspective, it associates the status of social representations with ergonomic features of the interface as variables moderating persuasion and intention in the context of ICT expansion. In particular, we reasoned that individuals can be persuaded if the argument of the message resonates with them, and also showed that the relevance of that message can be strengthened by the interface layout. We provided a theoretical conceptualization and empirical investigation of the above-mentioned moderating effects and applied them to organ donation. However, they can also be applied to other contexts. From a practical point of view, the progress made in this study can benefit all those seeking the large-scale dissemination of public information. In particular, this study recommends applying the structural approach to social representations in experimental designs involving persuasive technology. At the same time, it recommends taking the characteristics of the interface layout in account, in order to improve the impact of the message (i.e., salience of the central elements of the social representations) and the achievement of the persuasive goals. We hope that the results of this study will inform the design of persuasive interfaces and informative websites for social marketing in fields such as health promotion and environmental protection. Designing suitable technology can thus enhance persuasive effects by encouraging people to use the central route [20], and promote decision-making [60] by providing individuals with a shortcut to action [64].

## Perspectives

In any future application context, several avenues can be pursued to improve the impact of the message and the achievement of the persuasive goals. First, we recommend starting with the personalization of the message content. Customized messages are assumed to stimulate greater cognitive activity [111]. With reference to the sociodynamic model of social representations [112], it is possible to focus on the most cognitively salient elements of the representations for specific groups or individuals. For example, in the context of organ donation, Schultz, Nakamoto, Brinberg, and Haes [113] argued that general campaigns on this subject are not sufficient. In addition, several studies have shown the relevance of adapting communication to the target audience in organ donation [114–117]. We therefore argue that personalization, based on the sociodynamic model of social representations [112], can strengthen cognitive economy and provide a shortcut to action [64]. Second, it would also be interesting to study individuals’ motivation or capacity. A meta-analysis confirmed the relevance of using self-efficacy as a universally valid and reliable construct [118]. Believing in one’s capacity to accomplish difficult tasks is directly linked to behavior change. For example, beliefs such as outcome expectancies play an explicit and primary role in explaining motivation [119–121]. Bandura [122] argued that individuals engage in behaviors that give them satisfaction and self-esteem. Therefore, motivation can be improved by helping people to see how behavior change is in their own personal interest and can help them meet their personal goals. If this research area is linked to persuasive technology, made more relevant through personalization or tailoring (e.g., [123–125]), people can be provided with relevant information that allows them to achieve their personal goals and guides their behaviors. According to Bandura [121], positive verbal persuasion (e.g., encouragement) is a source of information that helps to build self-efficacy. Thus, with technological persuasion techniques, especially tailored or personalized persuasion, individuals can acquire greater self-efficacy. Additionally, high self-efficacy levels lead people to examine the information or arguments more carefully, and process it following the central route, thereby strengthening their initial confidence in their thinking [126]. This same self-efficacy could therefore allow them, for example, to speak more easily, acquire interpersonal skills, and even experience less social anxiety [127]. Finally, it would be useful to study the cognitive dissonance process [128, 129], exploring what kind of dissonance is at work in a context of persuasive technology, especially when people are exposed to counterattitudinal content. To our knowledge, no empirical studies have so far investigated the link between cognitive dissonance and persuasive technology. Veer and Shankar [130] focused on strategies for reducing dissonance in the context of persuasive technology through the justification-suppression model [131]. However, they did not provide any evidence of dissonance at work. It would be especially interesting to find out the nature and extent of the dissonance that is aroused.

## Supporting information

S1 Appendix Supporting procedure (preliminary study). (DOCX)

S2 Appendix Copy of the questionnaire (main study). (DOCX)
