# Supplementary material for: Social representations and interface layout: A new way of enhancing persuasive technology applied to organ donation
Source: PLoS One. 2020 Dec 31;15(12):e0244538. doi: 10.1371/journal.pone.0244538 (PMC7775091; doi:10.1371/journal.pone.0244538)
Supplement: S1 Appendix — (DOCX) [file pone.0244538.s001.docx]

In accordance with the TCI methodology and with previous studies [48,49,98,99], we calculated a percentage of centrality for each element of the social representations. Thus, the “yes” answers (i.e., 3 = *Generally yes* and 4 = *Definitely yes*) were grouped together and coded 1, while the “no” answers (i.e., 1 = *Generally no* and 2 = *Definitely no*) were grouped together and coded 0. Next, for the percentage of centrality, we calculated the ratio of “yes” answers to the total number of participants for each item, and multiplied this ratio by 100. Finally, each percentage of centrality for each representational element was compared with a decisional threshold, using the *D*max Kolmogorov‑Smirnov test [48,132]. This test makes it possible to identify items with a percentage of centrality that differs significantly from 100%. All items with a percentage of centrality that was greater than or equal to this threshold, were thus deemed to be central elements of the social representations. Participants considered that these elements were always, in every case, linked to the object of the social representations (i.e., organ donation). Therefore, according to Abric [44,45], these elements belonged to the central core because they were independent of the immediate context (see also [48]). All items with a percentage of centrality below the value of the threshold were deemed to be peripheral elements of the social representations. Their link to the object depended on the context.

**References**

1. Kandji, GK. 100 Statistical Test. New Delhi: Sage publications; 1999.
