# Supplementary material for: Social representations and interface layout: A new way of enhancing persuasive technology applied to organ donation
Source: PLoS One. 2020 Dec 31;15(12):e0244538. doi: 10.1371/journal.pone.0244538 (PMC7775091; doi:10.1371/journal.pone.0244538)
Supplement: S2 Appendix — (DOCX) [file pone.0244538.s002.docx]

**Copy of the questionnaire**

**(in original language)**

**1- A quel point vous sentez-vous satisfait(e) des diapositives qui vous ont été présentées ? Veuillez situer votre réponse sur une échelle de 0 à 10, 0 correspondant à « pas du tout satisfait », 10 correspondant à « tout à fait satisfait ».**

Tout à fait satisfait

Pas du tout satisfait

| 0 | 1 | 2 | 3 | 4 | 5 | 6 | 7 | 8 | 9 | 10 |
| --- | --- | --- | --- | --- | --- | --- | --- | --- | --- | --- |

**2-** **En ce moment, sur une échelle de 0 à 10, à quel point diriez-vous que vous êtes favorable au don d'organes ? (0 correspondant à « pas du tout favorable », 10 correspondant à « tout à fait favorable ») ?**

Tout à fait satisfait

Pas du tout satisfait

| 0 | 1 | 2 | 3 | 4 | 5 | 6 | 7 | 8 | 9 | 10 |
| --- | --- | --- | --- | --- | --- | --- | --- | --- | --- | --- |

**3- A quel point diriez-vous que vous êtes certain de la réponse que vous avez donné à la question précédente ? 0 correspond à « pas du tout certain », 10 correspond à « tout à fait certain ».**

Tout à fait satisfait

Pas du tout satisfait

| 0 | 1 | 2 | 3 | 4 | 5 | 6 | 7 | 8 | 9 | 10 |
| --- | --- | --- | --- | --- | --- | --- | --- | --- | --- | --- |

**4- A quel point diriez-vous que le don d'organes est quelque chose d'important pour vous ? 0 correspond à « pas du tout important », 10 correspond à tout à fait important ».**

Tout à fait important

Pas du tout important

| 0 | 1 | 2 | 3 | 4 | 5 | 6 | 7 | 8 | 9 | 10 |
| --- | --- | --- | --- | --- | --- | --- | --- | --- | --- | --- |

**5- Sur une échelle de 0 à 10, à combien estimeriez-vous à présent votre intention de vous déclarer donneur d'organes ? 0 correspond à « je n'ai pas du tout l'intention de me déclarer comme donneur d'organes », 10 correspond à « j'ai tout à fait l'intention de me déclarer comme donneur d'organes ».**

J’ai tout à fait l’intention

Je n’ai pas du tout l’intention

| 0 | 1 | 2 | 3 | 4 | 5 | 6 | 7 | 8 | 9 | 10 |
| --- | --- | --- | --- | --- | --- | --- | --- | --- | --- | --- |

**6- A présent nous vous demandons de lister toutes les pensées qui vous sont venues à l'esprit lorsque vous avez regardé les diapositives lors de la première phase de cette expérience. Écrivez vos pensées comme dans l'exemple ci-dessous. Vous pouvez écrire autant de pensées que vous souhaitez. Enfin, nous vous demandons d'être honnêtes et de lister tout ce qui vous est passé par la tête à la visualisation des diapositives.**

**
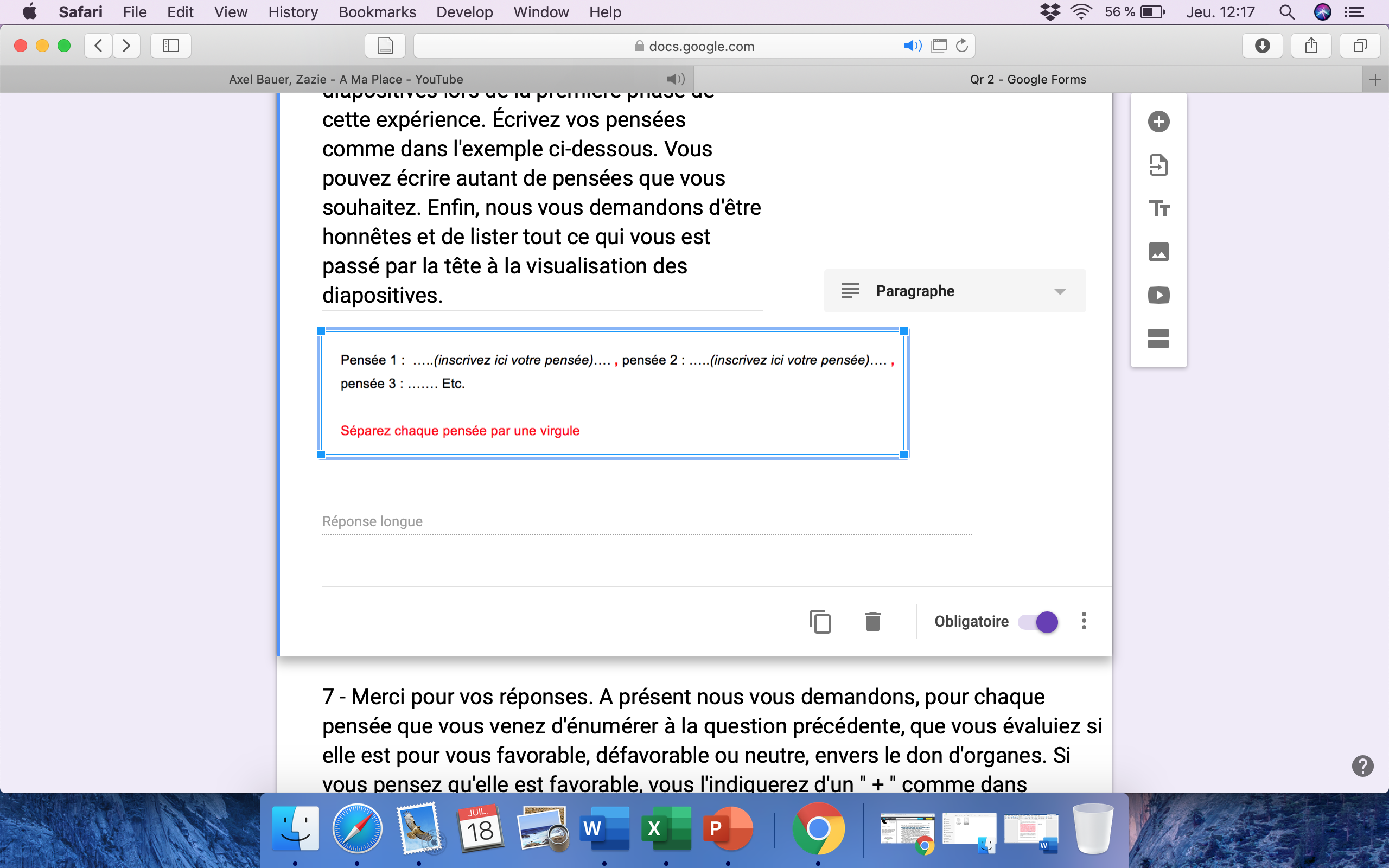
**

…………………………………………………………………………………………………..

**7 - Merci pour vos réponses. A présent nous vous demandons, pour chaque pensée que vous venez d'énumérer à la question précédente, que vous évaluiez si elle est pour vous favorable, défavorable ou neutre, envers le don d'organes. Si vous pensez qu'elle est favorable, vous l'indiquerez d'un " + " comme dans l'exemple ci-dessous. Si vous pensez qu'elle est défavorable, vous l'indiquerez d'un "-". Si vous pensez qu'elle est neutre, vous l'indiquerez d'un " 0 ".**

**
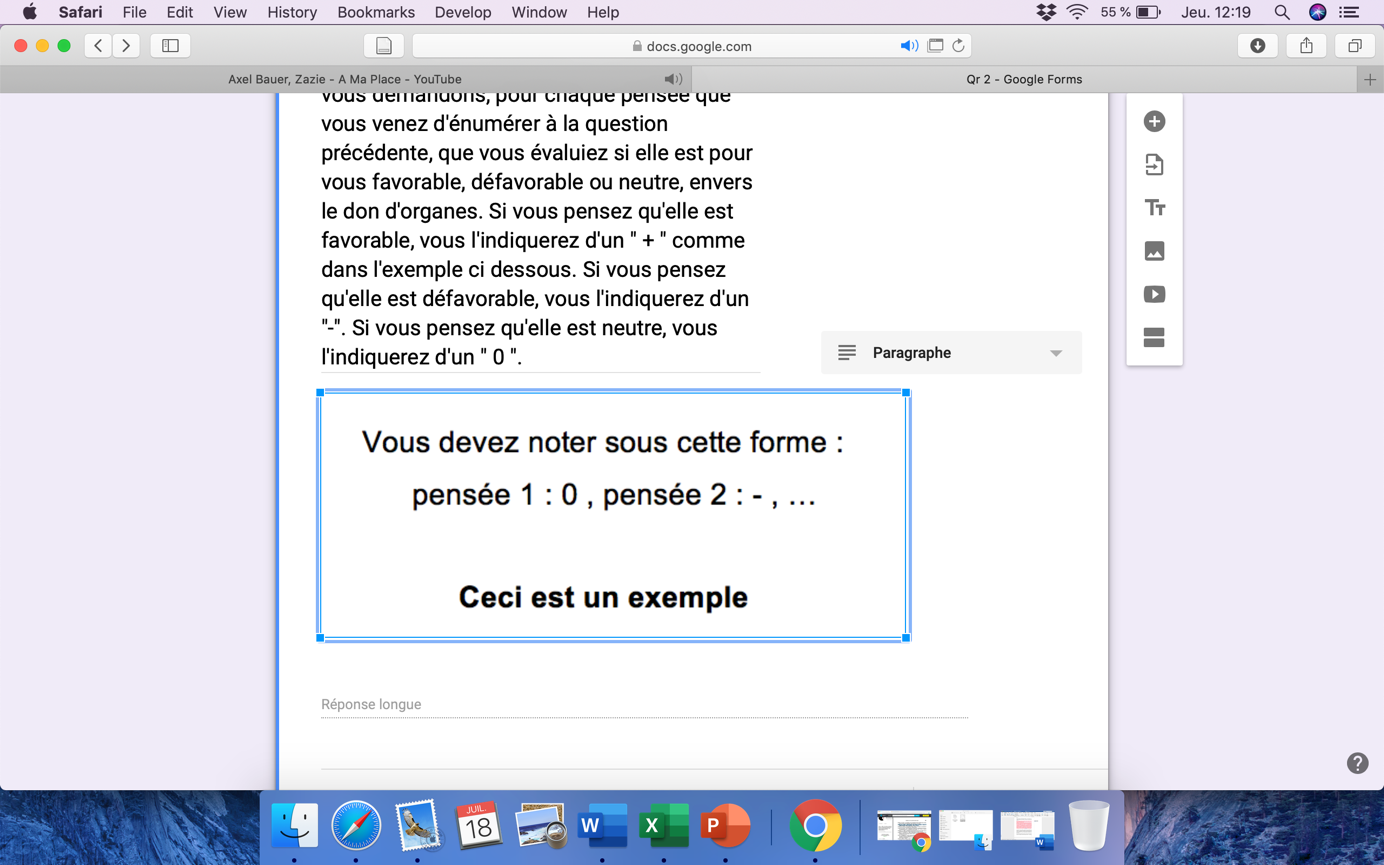
**

…………………………………………………………………………………………………..

**8- Pourriez-vous maintenant dire que vous étiez confiant lorsque vous avez eu ces pensées ? Veuillez situer votre réponse sur une échelle de 0 à 10, 0 correspondant à « pas du tout confiant », 10 correspondant à « tout à fait confiant ».**

Tout à fait confiant

Pas du tout confiant

| 0 | 1 | 2 | 3 | 4 | 5 | 6 | 7 | 8 | 9 | 10 |
| --- | --- | --- | --- | --- | --- | --- | --- | --- | --- | --- |

**9- Parmi les mots qui vous ont été présentés sur les diapositives de la tâche précédente, veuillez noter ceux dont vous vous rappelez, et ce dans l'ordre dans lequel vous vous en rappelez. Merci de séparer chaque mot par une virgule.**

…………………………………………………………………………………………………..

**10- Veuillez maintenant classer ces mots par ordre d'importance, c'est-à-dire des mots que vous jugez comme étant les plus importants relatifs au don d'organes (parmi ceux que vous avez cités) aux mots que vous jugez comme étant les moins importants relatifs au don d'organes. De même, veillez à séparer chaque mot par une virgule.**

…………………………………………………………………………………………………..

**11- Vous êtes :**

⃞ Un homme

⃞ Une femme

**12- Ecrivez votre âge :**

…….. ans

**13- Etes-vous greffé d'un ou plusieurs organes ?**

⃞ Oui

⃞ Non

**14- Connaissez-vous dans votre entourage des personnes ayant reçu une greffe d'organes ou étant en attente d'un organe ?**

⃞ Oui

⃞ Non

**Copy of the questionnaire**

**(in English)**

**1- How satisfied are you with the slides presented to you? Please rate your answer on a scale of 0 to 10, with 0 being "not satisfied at all " and 10 being "completely satisfied".**

Completely satisfied

Not satisfied at all

| 0 | 1 | 2 | 3 | 4 | 5 | 6 | 7 | 8 | 9 | 10 |
| --- | --- | --- | --- | --- | --- | --- | --- | --- | --- | --- |

**2- At this time, on a scale of 0 to 10, how would you rate your support for organ donation (0 being "not in favour at all", 10 being "very in favour")?**

Very in favour satisfied

Not in favour at all

| 0 | 1 | 2 | 3 | 4 | 5 | 6 | 7 | 8 | 9 | 10 |
| --- | --- | --- | --- | --- | --- | --- | --- | --- | --- | --- |

**3- How certain would you say you are of the answer you gave to the previous question? 0 corresponds to "not certain at all", 10 corresponds to "quite certain".**

Quite certain satisfied

Not certain at all

| 0 | 1 | 2 | 3 | 4 | 5 | 6 | 7 | 8 | 9 | 10 |
| --- | --- | --- | --- | --- | --- | --- | --- | --- | --- | --- |

**4- How important would you say organ donation is to you? 0 means "not important at all", 10 means "absolutely important".**

Absolutely important

Not important at all

| 0 | 1 | 2 | 3 | 4 | 5 | 6 | 7 | 8 | 9 | 10 |
| --- | --- | --- | --- | --- | --- | --- | --- | --- | --- | --- |

**5- On a scale from 0 to 10, how would you rate your intention now to declare yourself as an organ donor? 0 corresponds to "I do not intend to declare myself as an organ donor at all", 10 corresponds to "I fully intend to declare myself as an organ donor".**

I fully intend

I do not intend at all

| 0 | 1 | 2 | 3 | 4 | 5 | 6 | 7 | 8 | 9 | 10 |
| --- | --- | --- | --- | --- | --- | --- | --- | --- | --- | --- |

**6- Now we ask you to list all the thoughts that came to your mind when you looked at the slides during the first phase of this experience. Write your thoughts as in the example below. You can write as many thoughts as you want. Finally, we ask you to be honest and list everything that came to mind when you viewed the slides.**

Thought 1 : ….(enter your thought here)…. , thought 2 : ….(enter your thought here)…. , thought 3 : …. Etc.

Separate each thought with a comma

…………………………………………………………………………………………………..

**7 - Thank you for your answers. Now we ask you, for each thought you have just listed previously, to evaluate whether it is favourable, unfavourable or neutral for you towards organ donation. If you think it is favourable, you will indicate it with a "+" as in the example below. If you think it is unfavourable, you will indicate it with a "-". If you think it is neutral, you will indicate it with a "0".**

You must note in this form:

Thought 1: 0 , thought 2: - , ...

**This is an example**

…………………………………………………………………………………………………..

**8- Now, could you say that you were confident when you had these thoughts? Please rate your answer on a scale of 0 to 10, with 0 being "not confident at all " and 10 being "quite confident".**

Quite confident

Not confident at all

| 0 | 1 | 2 | 3 | 4 | 5 | 6 | 7 | 8 | 9 | 10 |
| --- | --- | --- | --- | --- | --- | --- | --- | --- | --- | --- |

**9- Among the words that were presented to you on the previous task’s slides, please note the ones you remember, in the order in which you remember them. Please separate each word with a comma.**

…………………………………………………………………………………………………..

**10- Please, now, rank these words in order of importance, that is to say from the words you consider to be the most important related to organ donation to the words you consider to be the least important related to organ donation. Also, be sure to separate each word with a comma.**

…………………………………………………………………………………………………..

**11- You are:**

⃞ A man

⃞ A woman

**12- Write your age:**

............. years old

13- Are you transplanted from one or more organs?

⃞ Yes

⃞ No

**14- Do you know people around you who have received an organ transplant or are waiting for an organ?**

⃞ Yes

⃞ No
